# Temperamental differences in the Subtypes of Attention Deficit Hyperactivity Disorder

**DOI:** 10.1192/j.eurpsy.2024.373

**Published:** 2024-08-27

**Authors:** G. Longo, M. Servasi, L. Orsolini, U. Volpe

**Affiliations:** Dipartimento di Neuroscienze Cliniche/DIMSC, Università Politecnica delle Marche, Ancona, Italy

## Abstract

**Introduction:**

Attention-Deficit/Hyperactivity Disorder (ADHD) is a neurodevelopmental condition marked by difficulties in attention, hyperactivity, and impulsivity. Its subtypes—predominantly inattentive, predominantly hyperactive-impulsive, and combined—vary in symptom presentation and impact on daily functioning. Understanding these subtypes is crucial for tailored interventions and support.

**Objectives:**

Our aim is to clinically characterize the psychopathological aspects of the subtypes of ADHD.

**Methods:**

Our study is conducted on patients (>18 years) referred to the adult ADHD outpatient service of the Psychiatric Clinic of Ancona (Università Politecnica delle Marche, Italy). The Diagnostic Interview for ADHD in adults (DIVA 5.0) was used for diagnosing ADHD. The following rating scale were administered: Temperament Evaluation in Memphis, Pisa and San Diego (TEMPS-M), and Temperament and Character Inventory-Revised (TCI-R).

**Results:**

76% (n=170) of all screened patients were diagnosed with ADHD in adulthood. 57.6% (n=98) were diagnosed with ADHD combined subtype, 35.3% (n=60) with ADHD inattentive subtype, and 7.1% (n=12) with ADHD hyperactive subtype. Only 12.9% (n=22) were diagnosed with ADHD in childhood. Based on the results obtained at TEMPS-M, 43.8% (n=32) of patients were found to have cyclothymic temperament. Subjects with ADHD combined subtype scored significantly higher mean on the irritable temperament subscale of the TEMPS-M than those with ADHD inattentive subtype (p=0.016), while patients with ADHD inattentive subtype had a significantly higher mean score on the disorderliness subscale of the TCI-R than those with ADHD hyperactive and combined subtype (p=0.010). Given the logistic regression analyses using the TCI-R, developing an inattentive type of ADHD is negatively predicted by the disorderliness subscale of the TCI-R (exp(B)=0.788, IC95%=0.669-0.929, p=0.005) and positively predicted by the extravagance subscale of the TCI-R (exp(B)=1.104, IC95%=1.009-1.208, p=0.031), the hyperactive subtype of ADHD is negatively predicted by the fatigability subscale of the TCI-R (exp(B)=0.775, IC95%=0.597-1.005, p=0.055) and the combined subtype that is positively predicted by the disorderliness subscale of the TCI-R (exp(B)=1.140, IC95%=1.011-1.287, p=0.033). Regarding temperament, through a logistic regression analysis, the inattentive subtype of ADHD is negatively predicted by the irritable temperament subscale of the TEMPS-M (exp(B)=0.904, IC95%=8.39-0.974, p=0.008), while for the combined subtype of ADHD it is positively predicted by the irritable temperament subscale of the TEMPS-M (exp(B)=1.088, IC95%=1.014-1.167, p=0.019).

**Conclusions:**

The results show that irritable temperament is a predictor for the inattentive and combined subtype, but with different polarities. In addition, how different patterns of personality are specific to the various subtypes of ADHD are highlighted.

**Disclosure of Interest:**

None Declared

